# Product selectivity and mass transport in levulinic acid transfer hydrogenation by monolithic MIL-100, MIL-88B and ZIF-8@Pd MOFs

**DOI:** 10.3389/fchem.2022.1087939

**Published:** 2023-01-12

**Authors:** Sean R. McIntyre, Paola A. Saenz-Cavazos, Elwin Hunter-Sellars, Daryl R. Williams

**Affiliations:** ^1^ Department of Chemical Engineering, Imperial College London, London, United kingdom; ^2^ Lawrence Livermore National Laboratory, Livermore, CA, United States

**Keywords:** mass transfer, MOF, hydrogenation, zero-length column, biomass conversion, diffusion constant, stability

## Abstract

The diffusion processes between adsorbent and adsorbate naturally play a significant role in the efficiency and selectivity of the heterogenous catalytic process. This paper considers the importance of diffusion processes in the transfer hydrogenation reaction of levulinic acid to γ-valerolactone by MIL-88B, MIL-100, and ZIF8@Pd monolithic catalysts. Over a period of five catalytic cycles, it was shown that the Fe-based catalysts can achieve similar conversions to the ZIF-8 supported Pd, with the only current limitation being the lower aqueous stability of these MOFs. Diffusion constants were calculated using the ZLC method, with micropore diffusion limitation found for ZIF-8 and MIL-100 monolithic frameworks at 2.7 x 10^−8^ and 4.6 x 10^−8^ cm^2^ s^−1^ respectively. This diffusion limitation was also confirmed by IR spectroscopy with an increasing concentration of C—H bands on the MOF substrate post-reaction. Mass transfer coefficients, also calculated by ZLC, revealed increased mass transport for the hydrophobic ZIF-8 framework, which perhaps aids in the γ-valerolactone selectivity over side products that are produced in the absence of catalytic material, as seen for MIL-88B and MIL-100 after multiple uses.

## Graphical Abstract

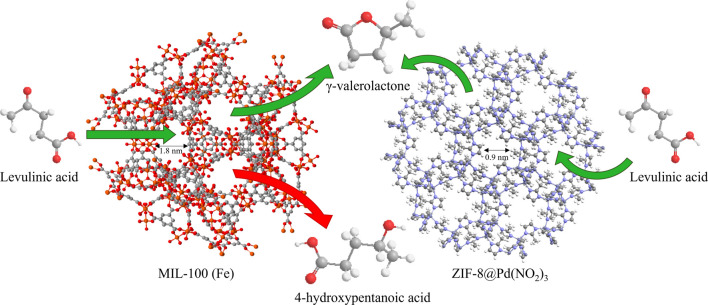

## 1 Introduction

One important research application for Metal-Organic Frameworks (MOFs) is the replacement of precious metals-based catalyst systems, especially in the synthesis of renewable fuels and sustainable platform chemicals from lignocellulosic bio-based feedstocks. Levulinic acid (LA) can be readily obtained by the acid hydrolysis of carbohydrates (Ghorpade et al., 1997), making it an ideal feedstock for the production of γ−valerolactone (GVL) a green solvent (Horváth, 2008) itself and a precursor for green fuels (Bond et al., 2010). The conversion of LA to GVL is typically performed using traditional supported metal heterogeneous catalysts, with benign solvents, such as water, and the use of hydrogen. The hydrogenation reaction of double-bonded groups began with the use of noble metal catalysts such as Pd/C (Zwicky and Gut, 1978), with both molecular hydrogen and isopropanol used as the hydrogen donors (Rao and Perlin, 1983; Galletti et al., 2012). More recently, Pd nanoparticles in a SiO_2_ support were used as a catalyst for the hydrogenation of LA to GVL, with 97.3% conversion (Yan et al., 2013). However, research has since been directed towards producing fewer Nobel-metal catalysts and more sustainable processes. To this objective, from a green chemistry perspective, the reaction is of high interest, with literature studies covering catalytic activity, selectivity, and lifetime. For liquid-phase biomass conversion, several investigations have been reported in the field of catalytic stability, as the nature of the reaction medium and the density of the biomass reagents increase the likelihood of deactivation and/or leaching of the catalytic metal or support material.

In this catalytic reaction and many others, support materials are used to increase the surface area of the catalyst, while decreasing the overall catalytic loading. These support materials are often porous zeolites, MOFs, or zeolitic imidazolate frameworks. Framework adsorbents are synthesised with a metal centre and organic linkers which coordinate to form a stable, uniform, porous structure. These support structures are often tuneable micro or mesoporous structures which can have an intrinsic catalytic surface chemistry of their own, without the need for additional catalytic loading. However, in some cases, the support structure is somewhat inert and contributes towards a reaction by way of selectivity. For heterogeneous catalytic reactions, the catalytic material may be held inside the pore structure of the support, which may limit which reactant molecules and selected isomers are able to reach the catalyst sites, or the products that are able to form from said reactants inside the network (Bidabehere et al., 2018).

The continuous flow catalytic transfer hydrogenation (CTH) using Ru/ZrO_2_ and Ru/TiO_2_ was performed by Genuino et a. (Genuino et al., 2020) using molecular hydrogen in water and dioxane, where a weight hourly space velocity (WHSV) of 2.4 $g_{feed}g_{cat}^{-1}h^{-1}$ allowed for a LA conversion of between 80% and 90%. Likewise, continuous flow CTH was performed by Lopez-Aguado et al. (Lopez-Aguado et al., 2020) using a Zr-modified dealuminated beta zeolite in a Meerwein–Ponndorf–Verley (MPV) reaction with IPA as the proton source, with a 90% GVL yield obtained over 20 days at 170°C. Vasanthakumar et al. (2020) outlined, in a batch process, the transfer hydrogenation reaction using MIL-88B, in the absence of molecular hydrogen, with again IPA acting as the proton source. This experiment was done in batch mode to a conversion of 99% over 15 h. The activity of MIL-88B was said to arise out of the metal-hydride-like interactions with the IPA.

MIL-88B, through its coordination chemistry, possesses unit cell swelling properties in different solvents and this is reported to occur *via* hydrogen bonding between the solvent and metal framework. MIL-100 retains a similar structure to MIL-88B; however, the MOF has a static mesoporous structure due to its tri-dentate coordination ligand (Han et al., 2017; Duan et al., 2020). This coordination also leaves a large number of coordinatively unsaturated metal sites (Horcajada et al., 2007; Hall and Bollini, 2020), so a direct comparison between MIL-88B and MIL-100 should yield interesting results regarding the reaction mechanism and support structure design.

Furthermore, whilst a number of reaction mechanisms are proposed for these processes, few undertake the work required to demonstrate them completely or understand the influences of various reaction steps on product selectivity and process efficiency. Considering the density and viscosity of biomass feedstocks (Lomba et al., 2011) used in heterogenous reactions, the contribution of mass transport to selectivity may be both important and measurable. One method by which the diffusion constant of a substrate may be calculated is the zero-length column (ZLC) method using a chromatographic experiment. Here a small sample is set to adsorption equilibrium with the substrate (adsorbate) and then desorbed *via* purging with an inert carrier phase; the resultant desorption profile may be used to determine the diffusion characteristics. Both micro and macropore diffusion constants may be calculated using the technique, where particles of varying particle radii and experiments at varying flowrates are used to determine inter-particle and equilibrium contributions respectively.

**FIGURE 1:**
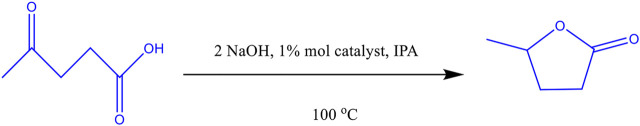
The reaction scheme for the levulinic acid transfer hydrogenation to γ− valerolactone.

In this current work, the transfer hydrogenation of levulinic acid to γ‒valerolactone in batch mode, Figure 1, using an assortment of MOFs and ZIFs, is investigated. The influence of pH on the reaction efficiency and selectivity is also reported. The diffusion constants of LA and GVL in IPA are observed for MIL-88B, MIL-100 and a relatively pore-restricted ZIF-8@Pd(NO_3_)_2_ using the ZLC method. These diffusion constants are then used to help explain the product selectivity and efficiency of the reaction process.

## 2 Materials and methods

### 2.1 Materials

All chemicals were purchased from commercial suppliers and used without further purification. Benzoic acid (98%), terephthalic acid (98%), trimesic acid (1,4-H_2_BDC, 95%), n-butylamine (99.5%), 2-methylimidazole (99%), zinc nitrate hexahydrate (>99%), pyridine (anhydrous, 99.8%), iron nitrate hexahydrate (98%), cyclohexane (>99%), levulinic acid (LA, 98%), γ-valerolactone (GVL 95%), palladium acetate (Pd(OAc)_2_ 98%), and acid washed glass-beads were obtained from Sigma-Aldrich. Sodium hydroxide (Fisher Scientific, 98.7%) isopropanol (IPA) (100%), DMF (100%), methanol (100%), ethanol (100%) and acetic acid (glacial 100%), were procured from VWR Chemicals. Iron chloride hexahydrate (FeCl_3_·6H_2_O) (97%–102%) was purchased from Alfa Aesar. Synthesised MOFs and ZIFs were synthesised in monolithic forms, without a requirement for shaping, MOFs were activated under vacuum (180°C for 12 h) before use.

#### 2.1.1 MIL-88B monoliths

For the MIL-88B synthesis FeCl_3_·6H_2_O (180 mg, 1.1 mmol), 1,4-H_2_BDC (120 mg, .72 mmol) with benzoic acid as a competing ligand (Yang et al., 2019) (22 mg, .18 mmol) in 6 mL DMF which was heated at 145°C for 1.5 h in solvothermal reaction. The collected product was washed with DMF, water and IPA, centrifuging at 6,000 rpm for 15 min, before the final drying and activation.

#### 2.1.2 ZIF-8@Pd(NO_3_)_2_ monoliths

A palladium (Pd) suspension of 280 mg palladium acetate particles and 580 mg of polyvinylpyrrolidone (PVP) acting as a stabilising agent, was mixed in 50 mL of methanol. .29 mL of 100% acetic acid was added to the solution containing palladium acetate prior to mixing with the zinc source, .745 g (∼2.5 mmol) of zinc nitrate hexahydrate. A second solution of 1.642 g (∼20 mmol) of 2-methylimidazole and 1.98 mL (∼20 mmol) of n-butylamine was created in 50 mL of methanol. When dissolved, the two solutions were mixed, inducing sol-gel formation (Hunter-Sellars et al., 2021). After 24–72 h, this mixture was then centrifuged for 10 min at 3500 rpm (to avoid sintering of particles), and then cleaned using methanol before the final drying and activation.

#### 2.1.3 MIL-100 monoliths

Low porosity MIL-100 was obtained following the procedures outlined in Kyong-Seo et al. (Seo et al., 2012), in which n-butylamine, 1.29 mL (∼13 mmol) was used as a capping reagent in the iron nitrate, 8.016 g (∼20 mmol), trimesic acid, 2.752 g (∼13 mmol), hydrothermal reaction (20 mL water, 160°C). After 12 h at 160°C, the mixture was cooled, and the product was obtained by centrifugation at 10 min at 3,500 rpm. The product was then cleaned using ethanol and D.I. water and allowed to dry before a final activation.

### 2.2 Physical characterisation

Fourier transform infrared (FT-IR) spectra were recorded using an Agilent Cary 630, between 500–4,000 cm^-1^. Extended FT-IR spectra may be found in the Supplementary Figures S7–9. X-Ray Diffraction (XRD) patterns were collected on a PAN analytical X’pert pro, (40 kV, 20 mA) with scans of 2θ over a range of 5–50. The material surface area and pore dimensions were determined using N_2_ adsorption/desorption isotherms using a Micrometrics 3Flex system with the Brunauer-Emmett-Teller (BET) method. In this work, chromium sputter coating (15 nm thickness) was applied before imaging by SEM. Images were collected on the SEM Zeiss Leo Gemini 1525, equipped with a field emission gun of 5 kV accelerating voltage.

#### 2.2.1 Catalysis methods

To assess the activity of the MOF and ZIF catalysts, reactions were carried out in batch and continuous flow modes. The post-reaction mixture was then passed through a .2 μm PTFE filter before being analysed using a GC-FID/MS (Shimadzu GC-2030/GCMS-QP2020 NX) equipped with a Shimadzu SH-Rxi-5ms column (30 m, .25 mm ID, .25 μm) Crossbond^®^ (5% diphenyl/95% dimethyl polysiloxane).

#### 2.2.2 Batch reaction

In a 10 mL round bottom flask, levulinic acid, 1 mmol, was added to 3 mL of isopropanol, along with 1 mmol NaOH and 3 mg of catalyst. TFH reactions were carried out at 100°C, continuously stirred (250 rpm), and refluxed for 15 h. Where pH measurements were taken, a Mettler Toledo (FiveEasy) pH probe was inserted into the reaction mixture at intervals over the course of the reaction. The pH was left to stabilise before taking any readings.

#### 2.2.3 Catalyst lifetime assessments

All three catalysts were reused for five cycles. Catalytic recycling was performed using the following process. Post-reaction, the catalyst was separated from the product mixture by centrifugation at 7,000 rpm for 20 min, with further centrifugation occurring after each washing step. The catalyst was then washed with IPA, twice with a 50/50 IPA/water mixture, and finally with IPA again before drying and reuse. Catalysts were activated initially under vacuum at 180°C, but not between cycles.

#### 2.2.4 Diffusion constant measurements

ZLC measurements were made using a Shimadzu Prominence HPLC, with the sample held within a re-fitted Phenomenex guard cartridge. Experiments were made using .1 mM of adsorbate in isopropanol (IPA). Following equilibrium and saturation of the adsorbent pores, the mobile phase was switched to pure IPA and the desorption curve was used to determine the counter-diffusion constants. Data was collected *via* an in-line UV/Vis detector and temperature control was carried out using the column oven, with measurements made at 20, 40, 60, and 80°C, and .5, and 1.0 mL/min flow rates to ensure there was consistency in diffusion time constants with varying flow rate (Brandani and Mangano, 2021). Blank measurements were taken using a column of non-porous glass beads, to correct for system effects. A revised ZLC model, Eq. 1, used in this work, has been made to account for fluid hold-up effects, either by dead volume or by adsorption interactions (Verbraeken et al., 2021):

$$\ln\left(\frac{C}{C_{0}}\right)=\ln\left(\frac{2L}{\beta^{2}{+\left(1-L+\gamma \beta^{2}\right)}^{2}+L-1+\gamma \beta^{2}}\right)-\beta^{2}\frac{D_{e}.t}{R_{p}^{2}}$$ (1)

A linear plot of ln(C/C_0_) against time in the long-time region yields a gradient of β^2^D_e_/R_p_^2^. The dimensionless ZLC parameter, L, which should be greater than 1, can be found *via* the intercept and confirmed using Eq. 2 (Brandani, 2016):

$$L=\frac{1}{3}\frac{U}{V_{s}}\frac{R_{p}^{2}}{K.D_{e}}$$ (2)

where, V_s_ is the solid volume, U is the mobile phase flow rate, K is the adsorption equilibrium factor, D_e_ is the effective diffusivity and R_p_ is the pellet radius.

Pyridine adsorption was carried out using a frontal analysis method, a break-through type uptake method, in cyclohexane (.1–10 mM), with a flow rate of .25 mL min^-1^. Comparisons were made to a non-porous glass bead column for dead volume correction.

#### 2.2.5 Mass transfer coefficients

Mass transfer coefficients were obtained for the substrate LA using the same experimental set-up as in the ZLC measurements. Following the method laid out by Brandani and Mangano (Brandani and Mangano, 2022), larger concentration changes were used, with adsorptive column saturation at 100 mM LA in IPA, followed by a switch to pure IPA. The adsorbed concentration was calculated *via* ZLC/frontal analysis using the same experimental set-up as ZLC, with dead volume measured using a non-porous glass bead column of identical particle size. Where the flow rate is low, adsorbed concentrations at any time may be found using, Eq. 3:

$$M_{s}\left(\bar{n}-{\bar{n}}_{0}\right)=\overset{t}{\underset{0}{\int }}{\left(Fc\right)}_{Adsorbent}dt-\overset{t}{\underset{0}{\int }}{\left(Fc\right)}_{Blank}dt$$ (3)

where, n_0_ = 0 in an adsorption experiment.

With the change in adsorbed concentration over time provided by a high flow rate experiment and Eq. 4:

$$M_{s}\frac{d\bar{n}}{dt}={\left(FC\right)}_{IN}-{\left(FC\right)}_{out}-V_{F}\frac{dc}{ct}$$ (4)

The mass transfer coefficient was found using Eq. 5:

k.a=dn¯dtneq−n¯ (5)

where, k is the mass transfer coefficient, a is the surface-to-volume ratio, and n_eq_ is the equilibrium adsorbed concentration, found from equilibrium adsorption isotherms, and $\bar{n}$ is the average adsorbed phase concentration.

## 3 Results and discussion

### 3.1 Synthesis and characterisation

In this paper three monolithic catalysts have been tested for catalytic activity; MIL-88B, MIL-100 and ZIF-8@Pd(NO_3_)_2_. These three catalysts all exhibit structural differences; MIL-88B possesses unit cell swelling properties, ZIF-8 possesses gate-opening behaviour, and MIL-100 is rigid and structurally fixed. MIL-100 and MIL-88B are isochemical, but not isostructural, both containing an Fe centre with aromatic carboxylic acid linkers. These materials were successfully synthesised in-house in a monolithic form, *via* framework modulation.

**FIGURE 2:**
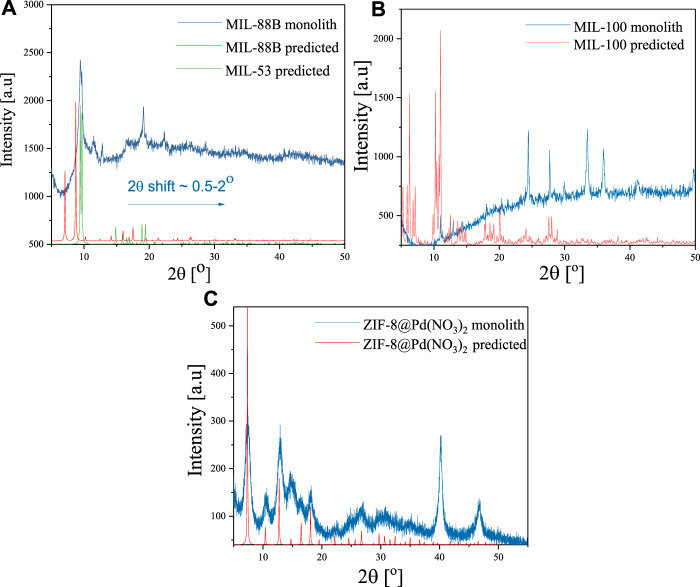
Experimental and predicted XRD spectra for monolithic **(A)** MIL-88B (Fe), **(B)** MIL-100 (Fe), and **(C)** ZIF-8@Pd(NO_3_)_2._

The XRD data agrees well with the literature and predicted diffraction patterns as shown in Figure 2. Some minor deviations in scattering angles attributed to the monolithic nature of these materials were observed (Hunter-Sellars et al., 2021). For the doped MIL-88B, a shift of 2θ ≈ .5–2 was observed, possibly due to the flexible unit cell (Horcajada et al., 2007), along with an overall decrease in crystallinity displayed in the peak broadness (9, 11.5, 12.9, 19.1, 22.4, 28.7). It is possible that the structure more closely resembles that of MIL-53, which may be made from the same ligand and metal centre under identical conditions; the diffraction pattern comparison is also shown in Figure 2A. A similar scattering angle shift was seen for the doped MIL-100, however the method of doping appears to have had a greater effect on the unit cell, with low scattering angles (Duan et al., 2020) almost completely absent, and an increase in larger scattering angles (10.9, 12.9, 14.4, 18.1, 20.4, 24.4, 27.7, 30, 33.^o^, 35.9). ZIF-8 @Pd(NO_3_)_2_ showed the highest crystallinity with almost all the scattering peaks present, with further peaks at 40.2 and 46.8 indicating the presence of Pd (Király et al., 1996; Suleiman et al., 2009).

**FIGURE 3:**
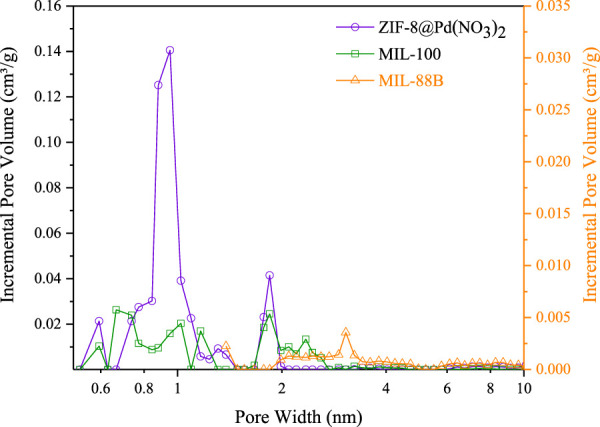
Pore size distributions calculated from BET–Tarazona NLDFT theory assuming cylindrical geometry, based on N_2_ adsorption data.

For MIL-88B and MIL-100 monoliths, FT-IR spectra, displayed peaks around 745 cm^-1^ (Fe-O or C-H), 1377 cm^-1^ and 1450 cm^-1^ (C=C or O-C-O) 1620 cm^-1^ (C=C), 1565 cm^-1^ (C=O). For the ZIF-8 monolith, peaks around 748 cm^-1^ (C=C bending), 1146 cm^-1^ and 1309 cm^-1^ (C-N), 1414–1711 cm^-1^ (C-H), and peaks around 2930 cm^-1^ (C-H stretch). N_2_ adsorption data, Figure 3; Table 1, revealed a disparity in surface area, confirming the influence of modulating ligands on the porosity, and therefore crystal growth. The MIL-100 monolith displayed relatively poor porosity, confirming the lack of crystallinity suggested by the XRD data. The ZIF-8 monolith retained decent porosity, perhaps suggesting that the capping agent n-butylamine has a less pronounced effect on basic ligands in porous frameworks. The small amount of MIL-88B microporosity observable by BET, is consistent with the literature values reported by Yurduşen et al. (Yurduşen and Yürüm, 2019).

**TABLE 1:** Catalyst material properties from N_2_ adsorption BET analysis.

Material	Pore diameter (nm)	BET surface area (m^2^g^−1^)	Total pore volume (cm^3^g^-1^)
ZIF-8@Pd(NO_3_)_2_	0.3^a^−0.9	902	0.49
MIL-100	0.7	51	0.16
MIL-88 B	3.1	215	0.23

^a^ Aperture at 0.3 nm was unobtainable by N_2_ adsorption, but is frequently reported in literature (Fairen-Jimenez et al., 2011).

### 3.2 Catalytic activity

#### 3.2.1 Substrate loading

During this investigation, it became apparent that overloading of the catalyst with LA substrate (>1%) prevented catalytic behaviour and lead to the formation of reaction side products. As this issue was ubiquitous for all catalysts, with a variety of pore distributions, it was assumed that this issue arose from the mass transfer of substrate to the catalyst, necessitating the need for the characterisation of mass transfer coefficients. This same decrease is observed by Wan et al. (2021) for a zirconium catalyst, but not to the same extent.

#### 3.2.2 Comparison of materials

**FIGURE 4:**
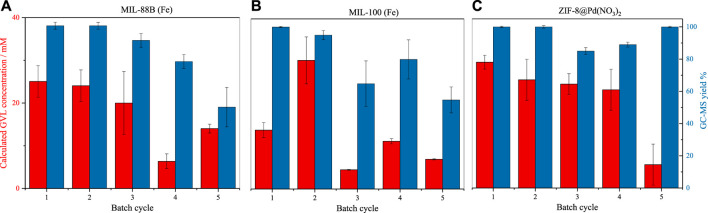
Batch transfer hydrogenation (100°C) of LA to GVL by catalysts **(A)** MIL-88B (Fe) **(B)** MIL-100 (Fe) **(C)** ZIF-8@Pd(NO_3_)_2_, assessed by GC-MS (errors taken from injection standard deviation, *n* = 3).

The catalytic conversion of levulinic acid is shown in Figures 4A–C. From these graphs, it can be observed that the catalyst pore size and surface area have little effect on the conversion, with ZIF-8 producing the highest conversion consistently. From the GC-MS yield, it is possible to infer that the smaller pore diameter imposes restrictions on side product formation, with the smaller pore aperture of ZIF-8 producing a product selectivity consistently above 80%. Where the pore apertures are larger, as in MIL-100 and MIL-88B, the selectivity decreases over time, which is due to the formation of local areas of high substrate concentration, or framework loss due to the basic conditions, producing the effect observed in substrate overloading. Surprisingly, the Fe-based MOFs performed comparably to the Pd catalyst but did not possess comparable material stability. For all materials, the conversion and subsequent turnover frequency (TOF) is low, especially when compared to like MOFs such as UiO-66(Zr) (Valekar et al., 2016), likely a result of low reaction temperature (Belguendouz et al., 2021).

#### 3.2.3 Catalyst stability

All three catalysts were reused for five cycles. Catalytic recycling was performed after washing and drying. Over the five cycles, all the reused catalysts showed a marked drop in conversion, and a decrease in selectivity was found for the Fe-based materials. In the case of MIL-100 the drop in reactivity over five cycles is significant, with a permanent decrease in conversion seen after two cycles. This sudden loss of reactivity is due to the hydrolytic breakdown of the hydrophilic framework, *via* base hydrolysis as a result of the high pH (Bezverkhyy et al., 2016), reducing the surface area and access to catalytic centres (López et al., 2021); see Supplementary Information for post-reaction (5 cycles) XRD. The same process likely occurs in MIL-88B, but at a lower rate. During the batch reactions, it becomes clear that the monoliths would slowly break up into smaller particles, due to stirring actions. The larger surface area of these smaller particles may make up for any surface loss activity, leading to the variations in activity from batch-to-batch (Vasanthakumar et al., 2020). Alternatively, the hydroxylation of the metal centres, in the presence of -OH, may hinder substrate-catalyst interactions, also contributing to variations in batch-to-batch activity. It is expected that some of the Fe-metal leached due to the relatively high pH. However, due to the lack of maintained catalytic activity (which would be expected if homogenous catalysis was occurring) it is supposed that these nanoparticles do not contribute towards the selective catalysis of GVL.

In the case of ZIF-8@ Pd(NO_3_)_2_, there is a slow decrease in reactivity, followed by a sharp decrease; it is theorised that this may be the result of Pd nanoparticle agglomeration or more likely substrate build-up resulting in pore blockages. A comparison of ZIF-8@Pd(NO_3_)_2_ XRD patterns pre- and post-reaction, Supplementary Figure S4, reveals the retention of Pd(NO_3_)_2_ peaks, ruling out significant leaching, suggesting pore blockages are the main contribution to the reduction in catalytic conversion. Any conclusions of Pd leaching by XRD here are qualitative, not quantitative.

**FIGURE 5:**
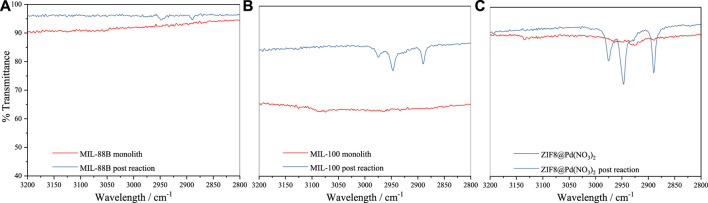
FT-IR spectra pre and post-reaction for **(A)** MIL-88B, **(B)** MIL-100, and **(C)** ZIF-8@Pd(NO_3_)_2._

To assess the possible build-up of a substrate on the surface, IR measurements were performed on the catalysts after five cycles, Figure 5, post-drying under ambient conditions.

### 3.3 Infra-red measurements

As shown in Figure 5 for the post-reaction materials, an increase in IR stretches between 2890–2980 cm^-1^ is observed. An increase in regions between 2850-2950 cm^-1^ normally relates to alkyl C-H stretching frequencies (Molpeceres et al., 2017), indicative of organic substrate build-up on the material surface. As shown, this build-up of organics follows the inverse of pore aperture diameter:

$$ZIF‐8@Pd{\left({NO}_{3}\right)}_{2}>MIL-100>MIL-88B$$

This indicates a possible mass transport/diffusion limitation imposed by the framework. These organics may be HPA, which is the precursor observed at low temperatures by Wei et al. (2022).

### 3.4 Reaction mechanism

**FIGURE 6:**
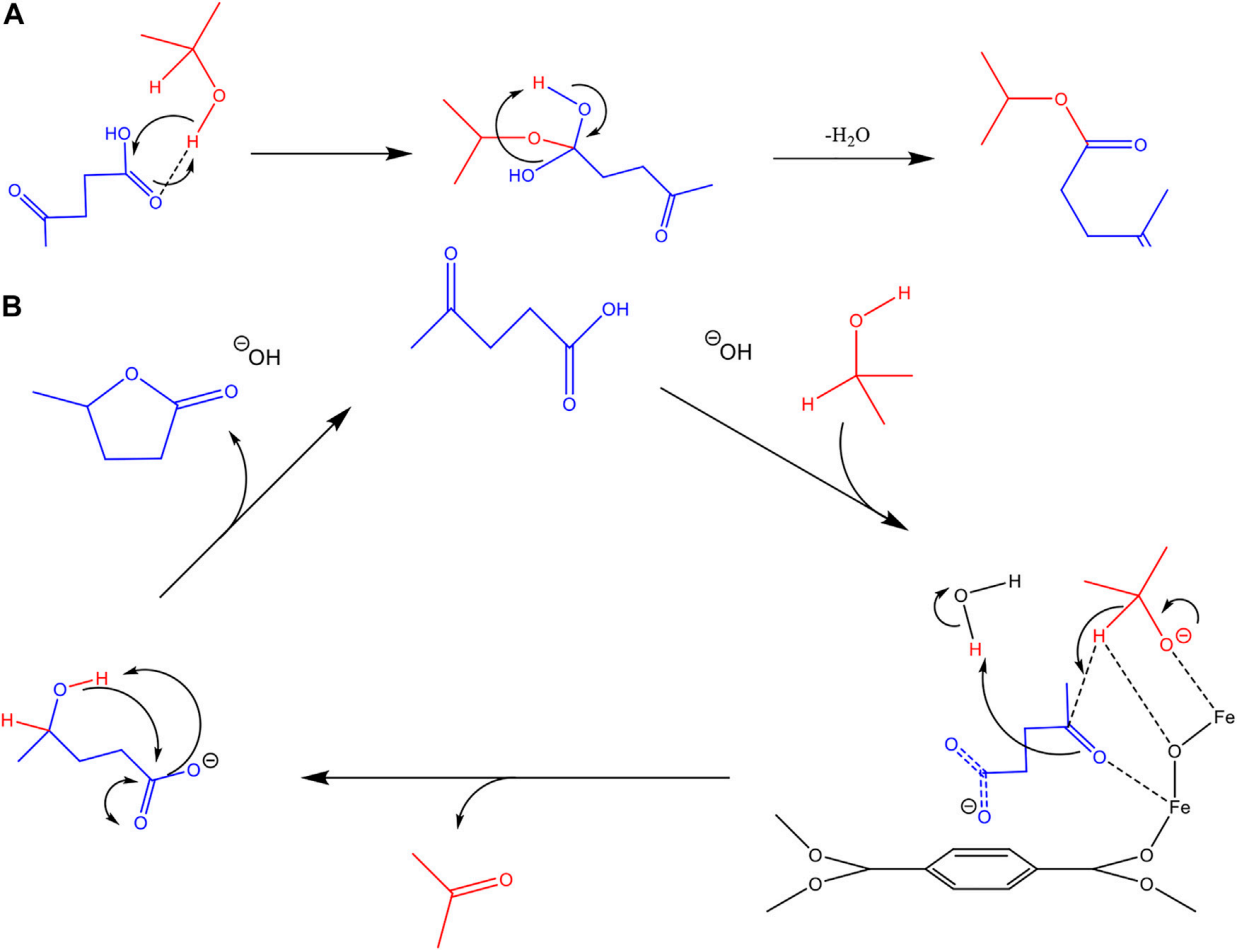
**(A)** Proposed conversion pathway for the side product isopropyl-levulinate, under basic conditions, absent of catalytic material. **(B)** A proposed reaction mechanism for the TFH of LA in the presence of active catalytic material, in this case MIL-88B.

Similar to that mechanism proposed by Vasanthakumar et al. (Vasanthakumar et al., 2020), a mechanism for the LA-TFH reaction using MIL-88 is outlined in Figure 6B. Under basic conditions, the isopropanol and LA are deprotonated and these both then interact with the metal centres of the MIL-88B framework. The transfer of hydrogen from isopropyl oxide produces acetone and the alcohol product which subsequently undergoes cyclisation to form γ−valerolactone (GVL). For all materials, where an equivalence of NaOH to LA, or high LA concentration (>1%) were used the reaction did not proceed to GVL and only yielded small amounts of the by-product shown in Figure 6A. With the NaOH equivalence, the pH did not exceed a value of 7, Supplementary Figure S1, which is not high enough for the abstraction of hydrogen from IPA. Under an excess of NaOH, 2 equiv., the pH reaches ∼13, high enough to create sufficient abstracted hydrogen to facilitate the transfer process.

In the case of substrate overloading, the product shown in Figure 6A is confirmed by GC-MS. It should be noted that this product was also found in a non-catalysed reaction, in lower abundance, suggesting a pathway absent of catalytic interaction. It is likely the localised build-up of LA on the catalytic surface promotes the formation of this impurity. In our experiments of 1% loading, no traces of this impurity were found, until after multiple cycles.

### 3.5 Zero-length column measurements

#### 3.5.1 Diffusion measurements

**FIGURE 7:**
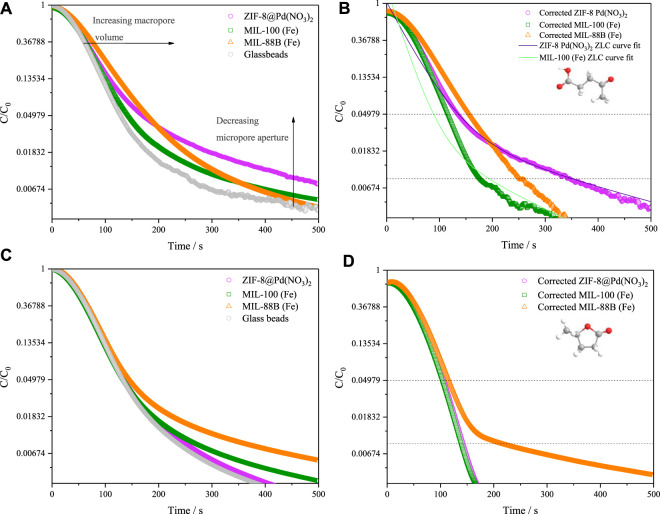
Liquid phase ZLC desorption curves for catalysts in IPA, shown for .5 mL min^-1^ at 80°C **(A)** levulinic acid, **(B)** levulinic acid corrected for sample geometry, **(C)** γ-valerolactone **(D)** γ-valerolactone corrected for sample geometry.

The effective diffusivities of LA and GVL in MIL-88B, MIL-100 and ZIF-8@Pd(NO_3_)_2_ were calculated using the ZLC method. This analysis allows the relationship to be determined between porosity and diffusion with catalyst lifetime and effectivity. Figures 7A, B shows the ZLC desorption curves for LA, with the calculated diffusivity values shown in Table 2. To ensure that the system noise did not contribute to the diffusion measurements, a range of C/C_0_ .01–.05 was used as the long-time region of analysis; any curves below this range are within the range affected by noise and therefore were discounted.

**TABLE 2:** Zero-length column experimental and modelled diffusion constants for LA and GVL at .5 mL min−^1^, 80°C.

Material	Diffusion constant/cm^2^ s^−1^ (Diffusion limitation)					
	levulinic acid			γ-valerolactone		
	Long-time apparent diffusion constant	ZLC modelled diffusion constant	Model uncertainty	Long-time apparent diffusion constant	ZLC-modelled diffusion constant	Experiment uncertainty
ZIF-8 @Pd(NO_3_)_2_	4.7 × 10^−8^ (micropore)	2.7 × 10^−8^	4.9 × 10^−10^	3.9 × 10^−7^ (not limiting)	N/D	2.9 × 10^−8^
MIL-100	8.8 × 10^−8^ (micropore)	4.6 × 10^−8^	3.2 × 10^−9^	4.0 × 10^−7^ (not limiting)	N/D	4.4 × 10^−8^
MIL-88B	1.2 × 10^−7^ (macropore)	N/D	N/D	6.6 × 10^−8^ (not limiting)	N/D	2.8 × 10^−9^

Where N/D is non-determinant due to poor model fit, or flow rate dependent diffusion constants.

The short-time region displays macropore diffusion contributions with:

$$ZIF-8@Pd{\left({NO}_{3}\right)}_{2}<MIL-100<MIL-88B$$

with MIL-88B having the largest macropore volume. The opposite may be said in the long-time region, between the dashed lines, where micropore control is observed as:

$$ZIF-8@Pd{\left({NO}_{3}\right)}_{2}>MIL-100>MIL-88B$$

with ZIF-8@Pd(NO_3_)_2_ having a smaller pore aperture. Unexpectedly, from Figures 7C, D the large GVL molecule shows no diffusion limitation in any of the materials. Therefore, as GVL may have a slightly larger critical diameter than LA, it is likely that any LA diffusion limitations arise increasing surface diffusion effects, facilitated by the much stronger interactions between catalyst and substrate, compared to catalyst and product (Yuta et al., 2009; Jakob et al., 2021). Again, for a reaction to be efficient the removal of products from the surface and tortuosity is important as it drives the reaction forward. Though the MIL-88B material does show some tailing, and a slightly lower apparent diffusion constant than the ZIF-8 of MIL-100, this tailing is not the result of kinetic limitation as the diffusion time constant varies with both flow rate and particle diameter. In the case of MIL-88B, any tailing in the desorption curve is the result of equilibrium control effects not being fully resolved by the blank subtraction.

From these diffusion constants, the FT-IR data observed in Section 3.2.8 can be rationalised. Since the diffusion of LA is slower in ZIF-8 and MIL-100, there is a greater build-up of molecules around the surface, when compared to MIL-88B, leading to the post-reaction IR alkyl bands observed. The slower diffusion of LA over GVL may explain why substrate overloading is observed as the LA concentration is increased beyond .5 mmol, with 1% catalyst loading.

#### 3.5.2 Mass transfer coefficients

**FIGURE 8:**
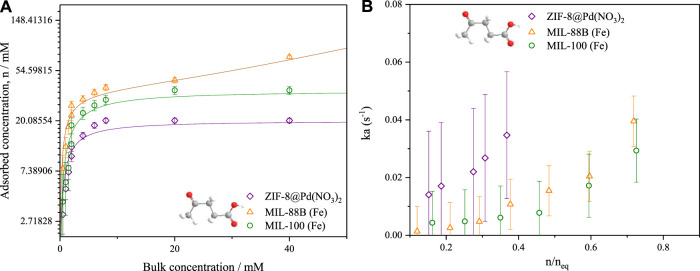
**(A)** LA/IPA liquid phase adsorption isotherms by low flowrate frontal analysis **(B)** associated mass transfer coefficients calculated using the high flowrate ZLC method. T = 80°C. The lines presented represent fitted linear Langmuir and modified BET isotherms.

Adsorption equilibrium isotherms for levulinic acid in IPA were calculated from low flow rate experiments, using the same ZLC set-up and a frontal analysis method. Figure 8A shows the relative shape of the isotherms with both microporous materials ZIF-8 and MIL-100 displaying type 1 isotherms, with MIL-88B displaying a type 2 isotherm owing to the increased meso- and macroporosity. From these isotherms the bulk concentration in a high flowrate, high concentration swing ZLC experiment may be related to adsorbed concentration, n_eq_. Further, the average adsorbed phase concentration, $\bar{n}$ , may be found over a set time, t, by determining the integral of the high flowrate experiments. Finally, dn/dt was found using Eq. 4, where the fluid phase volume, V_f_, and column mass, M_s_, is shown in Supplementary Table S1.

In Figure 8A, the isotherms were modelled using the Langmuir model in the case of both ZIF-8 Pd(NO_3_)_2_ and MIL-100; for MIL-88B, an adapted liquid phase BET model (Gocho et al., 1998; Girods et al., 2009) was applied due to the observable mesopore filling.

The resultant mass transfer coefficients, displayed in Figure 8B, show a concentration dependence which dramatically increases above n/n_q_ = .6. On average, the mass transfer coefficient for the ZIF-8 was larger than both MIL-100 and MIL-88B, owing to the increased surface area and framework hydrophobicity (Hunter-Sellars et al., 2021), see Table 3. The increased mesoporosity of MIL-88B will facilitate mass transfer more over the lower surface area of MIL-100. The barriers to mass transfer, such as surface defects and catalytic site-substrate interactions, may be found at the surface and have been cited as being influenced by surface heterogeneities (Hu et al., 2021). Therefore, it can be argued that although the ligand modulation increases the pore size distribution of a MOF, it can also produce surface defects that inhibit the entrance of molecules to the pores of crystals, increasing the contribution of surface diffusion toward the overall mass transfer process.

**TABLE 3:** Catalyst mass transfer coefficients for LA calculated by ZLC.

Material	Mass transfer coefficient, ka (s^−1^)	Experimental error (s^−1^)
ZIF-8 @Pd(NO_3_)_2_	0.035	0.022
MIL-100	0.011	0.010
MIL-88 B	0.015	0.008

From this mass transfer data, it is clear that the overall mass transfer of reactants into the catalyst is the crucial factor for product selectivity, where there are side reactions occurring in the bulk. As frameworks collapse over repeating cycles (for MIL-100 and MIL-88B), this mass transfer decreases further, further reducing GVL selectivity.

The calculated mass transfer coefficients **ka** were of a similar magnitude to those found by Grant Glover et al. (2008) for water in silica gel and hexane in activated carbon. And similar to those determined by Štěpánek et al. (2000) also for water vapour in silica gel.

#### 3.5.3 Base adsorption

**FIGURE 9:**
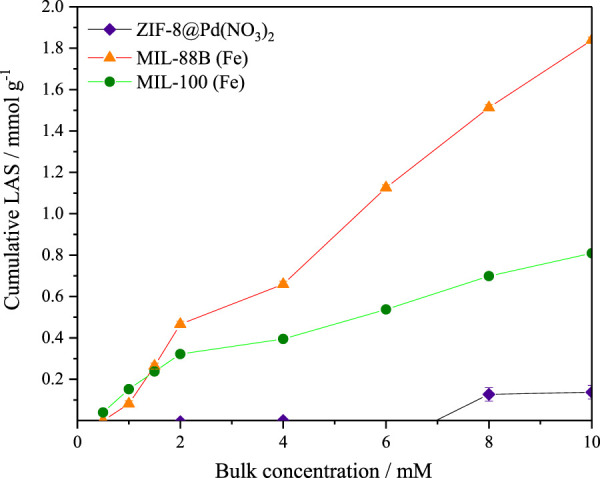
Adsorption of pyridine in cyclohexane by frontal analysis for the determination of acid site concentrations of ZIF-8@Pd(NO_3_)_2_, MIL-100, and MIL-88B. Errors taken from standard deviation (*n* = 3). Lines added for visualisation only.

It was found by Shao et al*.* that the acid and base site concentrations were important for LA to GVL reaction selectivity, with Lewis Acid sites (LAS) causing increased GVL ring opening (Shao et al., 2022). For this reason, the frontal analysis adsorption uptake of pyridine was measured. Assuming a 1:1 stoichiometry between the adsorbed pyridine and acid site (Fe, Zn, or Pd), the number for the total acid sites may be expressed in equivalent number of acid sites per unit area (mol equiv. g^-1^). The data from the pyridine uptake is shown in Figure 9, it is probable that the pyridine molecule is too large to enter the pores of the ZIF-8 monolith as the uptake is almost negligible. For MIL-100, the base uptake is initially higher than MIL-88B, due to the smaller pores improving uptake, however, the larger pore volume eventually leads to a higher observed uptake for MIL-88B. The larger number of LAS in MIL-88B was also reported by Yu et al. (2019). In all cases, it appears the isotherm plateau was approached, but not completed, and therefore the number of sites shown in Table 4 should be used for internal comparison, though it is unlikely the real value varies significantly.

**TABLE 4:** Experimental acid site equivalence by pyridine adsorption.

Material	Total amount of LAS/mmol g^−1^
ZIF-8@Pd(NO_3_)_2_	0.1
MIL-100	0.8
MIL-88B	1.8

From Table 4 it can be observed that the total equivalence of LAS calculated for the materials is also of a similar magnitude to those found Yu et al., in which the total LAS number was .24 and 4.93 mmol g^-1^ for MIL-100 and MIL-88B respectively.

It is likely that under the basic conditions provided by the NaOH, the total number of available LAS is decreased, leading to preferential GVL selectivity over the hydrogenolysis product 1,4-PDO (1,4-pentanediol).

In terms of dictating selectivity, there was no trend attributed to the increased Lewis acid sites and GVL product selectivity.

## 4 Conclusion

The batch conversion of levulinic acid (LA) to γ-valerolactone (GVL) was found to be successful for all three catalysts evaluated, with both Fe-based MOFs producing comparable conversion levels to the Pd catalyst. Upon assessing the material stability over multiple cycles, it became clear that the Fe MOFs were not as stable as the ZIF-8 framework, owing to the hydrophilic nature of the Fe framework, facilitating base hydrolysis by free OH^−^ ions.

In the case of MIL-100 and ZIF8@Pd(NO_3_)_2,_ the framework restrictions lead to some pore blocking or diffusion limitation, which was confirmed by both IR and the ZLC diffusion measurements.

ZLC experiments revealed that diffusion was dominated by Fe/Pd or hydrophobic interactions. For levulinic acid, the diffusion constant is likely affected by areas of locally increased LA concentration within the pores, caused by surface diffusion, with both the pore aperture and substate-catalyst interactions contributing to any limitations, with data following the trend MIL-88B > MIL-100 >ZIF-8@Pd(NO_3_)_2_. For GVL the diffusion appears to be influenced by the hydrophobicity, with diffusion being larger for the more hydrophobic ZIF material. In all cases the GVL diffusion was not restricted, possibly due to a decrease in interactions with the framework.

Assessments of mass transfer coefficients revealed that the materials with larger surface areas produced larger mass transfer coefficients, with ka values following .035 > .011>.006 for ZIF-8@Pd(NO_3_)_2_>MIL-88B>MIL-100. In general, these mass transfer coefficient trend well with product selectivity, which is understandable when considering that the side reactions occur in the absence of catalytic material. The GVL selectivity was driven by the presence of the MOF material framework, which for the Fe-based monoliths degrades over-time, resulting in lower activities and GVL yields.

## Data Availability

The original contributions presented in the study are included in the article/Supplementary Material, further inquiries can be directed to the corresponding author.
