# Supplementary material for: Product selectivity and mass transport in levulinic acid transfer hydrogenation by monolithic MIL-100, MIL-88B and ZIF-8@Pd MOFs
Source: Front Chem. 2023 Jan 12;10:1087939. doi: 10.3389/fchem.2022.1087939 (PMC9879703; doi:10.3389/fchem.2022.1087939)
Supplement: Supplementary file 1 [file DataSheet1.docx]

Product selectivity and mass transport in levulinic acid transfer hydrogenation by monolithic MIL-100, MIL-88B and ZIF-8@Pd MOFs.

Supplementary information

|  | ZIF-8@Pd(NO_3_)_2_ | MIL-88B | MIL-100 |
| --- | --- | --- | --- |
| Ms  (x10^-6^ kg) | 6.5 | 5.1 | 8.4 |
| n_eq_ | 20.18 | 45.05 | 36.84 |
| V_f_ (dm^3^) | 0.000144 | 0.000144 | 0.000144 |

*Table S1: Material properties required for mass-transfer calculations*

**Figure S1: A comparison of the effect of NaOH mole equivalence on measured pH during the batch transfer hydrogenation of levulinic acid. Error bars from repeats (n=3).**

**Figure S2: N_2_ adsorption isotherms for the ZIF-8@Pd(NO_3_)_2_, MIL-100 (Fe) and MIL-88B (Fe). Lines added for visualisation only.**

**Figure S3:** **A comparison of the XRD patterns for MIL-100 with low (1:4) and high (1:1) (n-butylamine:** **trimesic acid) ligand modulation ratios.**

**Figure S4: A comparison of the XRD patterns for ZIF-8@Pd(NO_3_)_2_ pre-reaction (bold) and post-reaction (fade). Pd(NO_3_)_2_ diffraction marked with dotted lines.**

**Figure S5: A comparison of the XRD patterns for MIL-88B pre-reaction (bold) and post-reaction (fade).**

**Figure S6: A comparison of the XRD patterns for MIL-100 pre-reaction (bold) and post-reaction (fade).**

**Figure S7: A comparison of the FT-IR spectra for ZIF-8@Pd(NO_3_)_2_ pre-reaction and post-reaction.**

**Figure S8: A comparison of the FT-IR spectra for MIL-100 pre-reaction and post-reaction.**

**Figure S9: A comparison of the FT-IR spectra for MIL-88B pre-reaction and post-reaction.**
